# Does Trophic Status Enhance or Reduce the Thermal Tolerance of Scleractinian Corals? A Review, Experiment and Conceptual Framework

**DOI:** 10.1371/journal.pone.0054399

**Published:** 2013-01-17

**Authors:** Katharina E. Fabricius, Szilvia Cséke, Craig Humphrey, Glenn De’ath

**Affiliations:** 1 Australian Institute of Marine Science, Townsville, Queensland, Australia; 2 Leibniz Center for Tropical Marine Ecology, Bremen, Germany; Swansea University, United Kingdom

## Abstract

Global warming, and nutrient and sediment runoff from coastal development, both exert increasing pressures on coastal coral reefs. The objective of this study was to resolve the question of whether coastal eutrophication may protect corals from thermal stress by improving their nutritional status, or rather diminish their thermal tolerance through the synergy of dual stressors. A review of previous studies on the topic of combined trophic status and heat exposure on the thermal tolerance of corals reveals a broad range of outcomes, including synergistic, additive and antagonistic effects. We conducted a 90-day long experiment exposing corals to realistic levels of elevated nutrients and sediments, and heat stress. Colonies of two common scleractinian corals (*Acropora millepora* and *Montipora tuberculosa*) were kept in coastal seawater, or coastal seawater that was further organically and nutrient enriched (OE), and/or enriched with nitrate. Batches of OE were created daily, facilitating nutrient uptake, plankton succession and organic enrichment as observed in coastal waters. After 10 days of acclimation, 67% of the colonies had their temperature gradually increased from 27° to 31.2°C. After 3–7 weeks of heat stress, colonies of both species had significantly greater reductions in fluorescence yields and lower survival in OE than without addition of OE. Furthermore, photophysiological recovery was incomplete 31–38 days after ending the heat stress only in the OE treatments. Nitrate alone had no measurable effect on survival, bleaching and recovery in either species. Skeletal growth rates were reduced by 45% in heat-stressed *A. millepora* and by 24% in OE-exposed *M. tuberculosa.* We propose a conceptual trophic framework that resolves some of the apparently contradictory outcomes revealed by the review. Our study shows that management actions to reduce coastal eutrophication can improve the resistance and resilience of vulnerable coastal coral reefs to warming temperatures.

## Introduction

Periods of high sea surface temperatures and light intensity have severe detrimental effects on scleractinian corals, causing bleaching, mortality and reduced calcification [1], [2]. Rising seawater temperatures from increasing atmospheric greenhouse gas concentrations not only increase the frequency and severity of heat stress periods, but also increase rainfall variability in many tropical regions [3], [4]. This leads to more severe drought-breaking or cyclone-induced floods, washing nutrients, sediments, and pollutants from cleared, fertilized and urbanized catchments into coastal waters [5], [6]. Both greenhouse gas concentrations and coastal development are predicted to continue rising significantly over the coming decades [7]. Reducing local stressors such as the loss of fertilizers and soils from farmed and cleared land is now considered the best management strategy to enhance the resilience of reefs to global warming [8], [9]. However, to assess the likely effectiveness of this strategy requires an improved understanding of the combined effects of thermal stress and terrestrial runoff on coral reefs.

The following review of the literature shows that it has remained equivocal as to whether corals exhibit synergistic, additive or antagonistic responses when simultaneously exposed to heat stress and elevated nutrients. Previous studies have used a wide range of field and controlled laboratory methods to investigate the issue, with treatments ranging from starvation in filtered water or removal of trace elements, to the addition of dissolved inorganic nutrients, suspended particulate matter, zooplankton or *Artemia salina* nauplii, and reduced water clarity. The studies suggest at least four different ways in which bleaching risk during temperature and insolation stress can be ameliorated in corals exposed to increased nutrients:

1. It is now clearly established that corals are more resistant (later onset of bleaching, longer tolerance of high temperatures before bleaching occurs, and milder symptoms) when they are well-fed rather than experimentally starved. For example, two species of temperature-stressed corals (*Stylophora pistillata* and *Galaxea fascicularis*) maintained higher photosynthetic efficiencies and cell division rates of algal endosymbionts (mitotic index) when fed with *Artemia salina* nauplii, while starved corals suffered progressive declines in photosynthetic efficiency and endosymbiont cell division rates [10]–[13].
2. Well-fed corals are also more resilient (having higher rates of survival and recovery from bleaching) compared to starved corals. Bleaching reduces photosynthetic carbon fixation, yet some species can prevent starvation by burning stored energy reserves, reducing metabolic rates, and/or increasing feeding rates (heterotrophic carbon gain) [14], [15]. For example, *Montipora capitata,* which has high energy storage and up-regulates heterotrophy when bleached, was found to recover faster from bleaching than two predominantly phototrophic species (*Porites lobata* and *P. compressa*) [16], [17]. Colonies of *Acropora intermedia* had lower bleaching and higher survival rates when supplied with suspended particulate matter compared to those not exposed to this source of food and shading [18], or when supplied with rotifers compared to colonies in filtered water [19].
3. The release of limitation by iron or other trace metals through terrestrial runoff may provide some protection against bleaching, by facilitating the generation of metalloenzymic antioxidants [20].
4. Corals may be more resistant to bleaching conditions on turbid inshore reefs, because clade D algal endosymbionts are usually found in turbid or low light environments on Indo-Pacific coral reefs [21], and these endosymbionts provide a ∼1°C higher temperature tolerance to many of their coral hosts compared to clade C endosymbionts [22] (but see also Point 4 below in the list of synergistic/additive effects).

In summary, according to these studies, corals in waters that are turbid, rich in nutrients and trace metals should be more resistant and resilient to temperature and solar insolation stress, since they have greater scope to maintain energy reserves through heterotrophy after endosymbiont loss, their antioxidant enzymes provide protection against oxygen radicals, and they may be equipped with more thermally tolerant endosymbionts.

There are however, at least six other lines of evidence based on physiological or correlative field data, which suggest synergistic or additive effects of temperature stress and nutrients. According to these studies, corals in nutrient-enriched or turbid waters are more vulnerable to temperature stress than those in low nutrient environments:

1. Corals exposed to high levels of dissolved inorganic nitrogen (DIN) experience greater bleaching susceptibility compared with corals in low nutrient environments. The proposed mechanism for this link is that high concentrations of DIN lead to high endosymbiont division rates, resulting in an increased demand for all essential plant nutrients by the proliferating endosymbiont populations, resulting in a relative under-supply of phosphate. The latter leads to altered thylakoid membrane structures with enhanced susceptibility to thermal and light damage [23].
2. Corals with high endosymbiont densities (e.g., as a consequence of high nutrient or iron supply [24]) also produce more harmful oxygen radicals compared to corals with low endosymbiont densities at the same level of heat stress [25]. Exposure to DIN can also reduce the photosynthetic efficiency and light-harvesting capacity of algal endosymbionts [26], possibly exacerbating heat damage to them. For example, gross photosynthesis in *Porites cylindrica,* standardized by pigment concentration or algal endosymbiont number, declined by ∼30% in corals exposed to either high NO_3_ or high temperature compared to controls, but declined by ∼60% in corals exposed to both high NO_3_ and temperature, suggesting an additive effect between both agents [27].
3. The increased photo-pigment densities of corals in a high-nutrient environment may also lead to greater temperature stress, as darker colony surfaces absorb more incident light energy than pale colonies [26], [28]. Darkly pigmented colony surfaces can be >1.0° warmer than the surrounding bulk seawater at high irradiance and low flow [28], [29], as is likely during the unusually calm and clear ‘doldrum’ conditions that often precede coral bleaching.
4. Although corals normally experience less solar insolation stress in turbid water [30], [31], shading by turbidity is typically diminished during bleaching conditions when waters tend to be unusually calm and clear. Hence the dark-acclimatized and darkly pigmented turbid-water colonies are likely to experience greater photoinhibition than light-acclimatized clear-water colonies during such bleaching conditions.
5. Spatial correlations between nutrient status and bleaching history suggest lower bleaching resistance per degree heating week on inshore compared with offshore reefs on the Great Barrier Reef during the 1998 and 2002 bleaching events [32], [33]. A similar relationship between bleaching extent and elevated chlorophyll a and DIN has been found in the Florida Keys [34]. The proposed mechanism is centered on DIN disrupting the trophic balance between algal endosymbionts and coral hosts at high temperatures and light.
6. Calcification in the massive *Montastraea faveolata* was reduced for longer after heat stress in turbid inshore compared to cleaner offshore reefs at similar levels of heat exposure (>8 years *vs.* 2–3 years) [35], [36].

The above review shows that from the existing literature and the theoretical perspective, it is surprisingly unclear as to whether corals exhibit elevated or reduced thermal tolerance when simultaneously exposed to heat stress and eutrophication. In this study, consisting of a 90-day long experiment, we aimed to expand our understanding of the link between chronic exposure of corals to turbid nutrient-enriched waters and their thermal tolerance from episodic heat stress. The objective of the experiment was to resolve whether coastal eutrophication may protect corals from thermal stress due to improved nutritional status [10]–[12], [16], [17], or rather increases their thermal sensitivity suggesting synergistic effects of these two forms of stress [27], [32], [33]. We investigated the temperature stress tolerance of two common Indo-Pacific coastal coral species, which were exposed for a prolonged period of time to environmentally relevant levels of nutrients and organically enriched sediments at ambient (outdoor) irradiance. Treatments simulated the chronic organical enrichment and plankton successions in coastal waters exposed to terrestrial runoff and sediment resuspension (rather than stress from episodic flood plumes with reduced salinity and peak concentrations of pollutants). Thermal tolerance was assessed by quantifying the survival, photophysiology and skeletal growth, before, during and after a period of thermal stress. We conclude with a conceptual framework on the effects of elevated nutrients or other forms of changes in water quality on the thermal stress tolerance in corals to synthesize and resolve the diverse outcomes of this and previous studies.

## Materials and Methods

### Study Species

Colony fragments were sourced from inshore reefs of the central GBR at 2–4 m depth. For the corymbose *Acropora millepora* (Ehrenberg, 1834), colony pieces were sourced from Pelorus (18°35′S, 146°31′E) and Double Cone Islands (20°07′S, 148°45′E), and branchlets were used to form nubbins (length ∼4 cm). For the foliose *Montipora tuberculosa* (Lamarck, 1816), colony pieces were sourced from Magnetic Island (19°10′S, 146°58′E), which were cut into ∼9–16 cm^2^ sized fragments. The water quality conditions of the collection sites are characterized in [37], [38]. The collection was approved as part of the 2007–2011 research plan of the Australian Institute of Marine Science (Great Barrier Marine Park Authority Permit-No: G09/30237.1).

Both study species are classified as highly susceptible to bleaching [39]. At the end of the experiment, samples were fixed in 100% ethanol and *Symbiodinium* types were determined based on sequence differences in the rDNA ITS2 region using denaturing gradient gel electrophoresis [40]. This analysis showed endosymbiont homogeneity, with *A. millepora* containing only C_2_ and *M. tuberculosa* only “C_1_-like” types of algal endosymbionts.

### Experimental Setup

The experiment was carried out at the Townsville site of the Australian Institute of Marine Science (AIMS). Light and water quality treatments were designed to be as environmentally relevant as possible. Twenty-four aerated 20 L glass tanks with flow-through seawater (4 L hr^−1^) were set up outdoors under a 30% light absorbing polycarbonate roof in three 1000 L water baths (8 tanks per water bath). Three Odyssey light loggers were used to record irradiance within the water baths (10-min readings throughout the 90 day period). The median daily maximum photosynthetic irradiance was 1017 µmol photons m^−2^ s^−1^ between 12∶00 and 13∶00 pm (range: 167–1340 µmol photons m^−2^ s^−1^), equivalent to a median of 25 mol photons m^−2^ d^−1^ (range: 5.3–50.7 mol photons m^−2^ d^−1^), depending on cloud cover and length of day. The tanks were supplied with 4 L hr^−1^ of coastal seawater (continuously pumped from the sea through a settlement tank and a 50 µm screen; salinity 33.5–35 PSU). A small bilge pump (10 W, Ascoll Powerhead 402) was placed into each tank to provide water flow and reduce particle settling. Six days before the experiment started, *A. millepora* nubbins were suspended in the water with nylon string from transparent plastic sticks, and four *M. tuberculosa* fragments were placed on the bottom of each tank (i.e. 16 colonies per treatment, a total of 96 nubbins per species). The water column over *A. millepora* and *M. tuberculosa* colonies was 10 and 16 cm deep respectively, hence differences in light exposure between enriched and unaltered seawater treatments and between colonies that were suspended and on the bottom were negligible (<3% difference at an estimated mean light diffusive attenuation coefficient K_d_ of 0.4 and 0.2).

On Day 1 of the experiment, corals were exposed to their respective nutrient and sediment treatments for acclimatization at ambient temperature. In summary, six treatments were established, each represented by four tanks, with four coral fragments of each of the two species added to each tank. Two levels each of temperature, organically enriched water and nitrate were used (as described in detail below): ambient temperature and heat stress (25°C vs. 31°C); without and with the addition of organically and nutrient enriched water (+OE); and without and with the addition of nitrate (+NO_3_). The ambient temperature treatments contained either Controls (ambient organic and nutrient concentrations of the coastal water) or +OE +NO_3_. Heat-stress treatments contained all four combinations of nutrient additions: Controls, +NO_3_, +OE, or +OE +NO_3_.

#### Temperature treatments

After 10 days of coral acclimatization to the water quality treatments at ∼27°C, the water in 16 of the 24 tanks (two of the three water baths) was gradually increased over a four-day period to 31.2°C (the mean long-term summer maximum temperature in the region is ∼30°C). Submersible titanium heaters were used to warm the water in the water bath, and large bilge pumps (5000 L hr^−1^) vigorously circulated the warmed water within the water baths to ensure uniform temperature across treatments. Water quality treatments were distributed evenly across these water baths (two of each treatment per water bath), and positions were randomised within water baths. Water temperature was measured daily in all tanks with a digital thermometer (accuracy ±0.1°C). Daily mean temperatures averaged 31.2°C ±0.6 SD in the heated tanks, and 25.3°C ±0.8 SD in the control tanks (range: 26.6°C ±0.6 SD in April to 24.6°C ±0.9 SD in July). The temperature was kept at these levels until the onset of severe decline in photosynthetic yields and visible bleaching in at least one treatment per species. This occurred after 23 days of heat stress for *A. millepora* and after 49 days for *M. tuberculosa*. Heat-stressed colonies from all treatments were moved into recovery tanks in the ‘ambient temperature’ water bath, with their exposure to NO_3_ and/or OE unchanged. The experiment was terminated after 38 days of recovery for *A. millepora,* and 31 days of recovery for *M. tuberculosa*.

#### Organically Enriched treatments (OE)

The coastal water around AIMS, located downstream of two major rivers (Burdekin and Haughton Rivers), contains substantial concentrations of nutrients and sediments (‘Control’ concentrations in Table 1), with a naturally high variability in particle and nutrient loads attributable to wind resuspension, river runoff and seasons [37], [38]. A 48-hr retention in large settlement tanks was used to dampen spikes in suspended solids. Typically, the control seawater appeared nitrogen limited compared with the Redfield ratio for nitrogen vs phosphorus of 16∶1 (molar ratios: 5.8 for DIN versus soluble reactive phosphorus, and 6.6 for particulate nitrogen versus particulate phosphorus; Table 1). Coastal sediment was sourced from the seafloor off AIMS from 2 m depth, sieved, and particles <350 µm were retained. The dry weight/volume ratio was determined, and 80 L was stored wet in sealed black drums in the shade. A new batch of 1000 L unfiltered seawater with coastal sediment and dissolved nutrients was made up daily, by adding both sediment and soluble plant fertilizer (Yates Thrive water soluble all-purpose plant food: N:P:K = 27∶5.5∶9; see below for final concentrations). The fertilizer choice was based on the fact that a large proportion of nutrients washed off agriculturally used catchments in north Queensland derive from plant fertilizers (albeit with molar ratios in the runoff varying between catchments and throughout the wet seasons [41]), and approximated Guillards f/2 enriched seawater medium formula designed to grow coastal marine algae [42]. To facilitate the development of nutrient-enriched plankton communities, each batch was incubated in a 1000 L tank (0.4 m deep) outdoors under the polycarbonate roof for 3–4 days before use. During this time, each batch was vigorously aerated and mixed by a large bilge pump (5000 L hr^−1^), however settlement of the larger particle fraction occurred. Two levels of organically enriched water (OE) were used: 12 tanks were supplied with 2 L hr^−1^ of the coastal seawater complemented with 2 L hr^−1^ of water from the incubation batch (+OE), and the remaining 12 tanks were supplied with the coastal seawater at 4 L hr^−1^, without OE addition.

**Table 1:** Seawater chemistry for the four treatments of organically and nutrient enriched water: Controls (unaltered coastal water), organical enrichment (+OE), and/or nitrate (+NO3, +OE +NO3).

	Control		+NO3		+OE		+OE +NO3	
*N*	12		6		7		13	
**TSS**	3.06	*(1.03)*	3.19	*(1.62)*	4.58	*(1.54)*	4.98	*(2.31)*
***N***	4		3		3		5	
**POC**	20.4	*(17.1)*	23.0	*(4.55)*	56.1	*(20.6)*	59.2	*(14.3)*
**PN**	2.50	*(1.69)*	3.63	*(0.49)*	7.82	*(1.85)*	7.79	*(2.67)*
**PP**	0.38	*(0.24)*	0.83	*(0.05)*	1.12	*(0.27)*	0.93	*(0.34)*
**Chl-a**	0.72	*(0.50)*	2.35	*(0.81)*	2.16	*(0.37)*	3.79	*(1.95)*
**DOC**	1.01	*(0.13)*	1.20	*(0.13)*	1.66	*(0.24)*	1.38	*(0.32)*
**NH_4_^+^**	0.21	*(0.01)*	0.22	*(0.01)*	0.21	*(0.01)*	0.21	*(0.02)*
**NO_2_^−^, NO_3_^−^**	0.23	*(0.23)*	0.26	*(0.13)*	0.24	*(0.19)*	0.12	*(0.08)*
**SRP**	0.076	*(0.019)*	0.058	*(0.002)*	0.115	*(0.040)*	0.073	*(0.023)*
**Si**	3.75	*(4.34)*	6.43	*(3.93)*	3.67	*(2.18)*	1.47	*(0.570)*

Mean values (±SD) of total suspended solids (TSS, mg L^−1^), particulate organic carbon (POC), nitrogen (PN) and phosphate (PP; all in µmol L^−1^); chlorophyll-a (Chl-a, µg L^−1^), dissolved organic carbon (DOC, mg L^−1^); dissolved inorganic nitrogen [ammonium (NH_4_+), nitrite and nitrate (NO_2_^−^,NO_3_^−^)]; soluble reactive phosphorus (SRP); and silicate (Si; all in µmol L^−1^). *N* = number of sampling occasions.

#### Nitrate treatments

Nitrate exposure was manipulated to distinguish between the commonly investigated direct effects of nitrate on the endosymbionts and the effects of organical enrichment on the coral holobiont. DIN rather than DIN plus phosphate was added, since an over-supply of DIN has been suggested to be largely responsible for declining thermal tolerance of corals [23], [43]. A 0.1 M KNO_3_ stock solution diluted with filtered seawater (0.0053∶ 1) was fed continuously into 12 tanks (+NO_3_: six with and six without OE) by means of a peristaltic pump (Masterflex L/S Digital Standard Drive, Cole-Parmer) with Tygon Tubing (3-stop; 2.06 mm id; Cole-Parmer) at a rate of 0.25 ml min^−1^. Nitrate in the +NO_3_ treatment was nominally increased by 4.0 µmol L^−1^ (but see below for uptake). The control tanks without nitrate addition had a mean ambient concentration of 0.23 µmol L^−1^.

The small pumps and aeration in each of the 24 tanks kept most of the particles suspended, however some settlement occurred especially in the corners of the tanks. Duplicate water samples were taken from the tanks to determine concentrations of total suspended solids (6 to 13 sampling dates), and dissolved and particulate nutrients and chlorophyll *a* (3 to 5 sampling dates; Table 1). Analytical protocols followed [38]. Enriched treatments contained ∼5 mg L^−1^ of suspended solids, with a 1.5- to 3-fold increase in particulate nutrients and ∼3–5-fold increase in chlorophyll compared with the controls in coastal seawater (Table 1). Final concentrations of the latter approximated or slightly exceeded the upper 95^th^ percentiles of values recorded on GBR inshore reefs such as Dunk and Magnetic Islands [38]. Concentrations of dissolved inorganic nutrients and total suspended solids were similar to those found on GBR inshore reefs in all treatments. Nitrate plus nitrite concentrations averaged ∼0.12 and 0.26 µmol L^−1^ in all treatments, despite a continuous NO_3_ addition to the +NO_3_ treatment tanks and 25% hr^−1^ water exchange rate (Table 1). This indicated rapid and almost complete biological uptake of NO_3_ by the productive coastal seawater used, as confirmed by the elevated concentrations of chlorophyll and particulate nutrients.

### Coral Responses

Differences in heat stress tolerance were quantified as differences in survival, photophysiological stress and recovery, and skeletal growth rates.

1. Survival of colonies was assessed daily, and mortality was defined as the point where tissue was sloughed off from >50% of the colony surface. Dying corals were removed from the tanks to avoid affecting other colonies. Tissue slothing, once started, was inevitably followed by the death of nubbins within 1–2 days. Survival was expressed as the proportion of colonies within each tank that survived to Day 71 of the experiment in *A. millepora*, and Day 90 in *M. tuberculosa*.
2. Photo-physiological responses were assessed using pulse amplitude modulation fluorometry (Imaging-PAM; WALZ, Germany). The effects of the different treatments on the photochemical capacity of photosystem II were explored by determining changes in chlorophyll fluorescence yields (F_v_/F_m_ = (F_m_ – F_0_)/F_m_ with F_v_, F_m_ and F_0_ being the variable, maximum and background fluorescence in dark-adapted state [44]. All colony fragments were dark-adapted for 30 minutes prior to each measurement in separate 20 L glass aerated tanks at experimental temperatures. Dark-adapted fragments were pulsed with a weak (<1 µmol m^−2^ s^−1^) red light to obtain F_0_, followed by a 1 s pulse of saturating actinic light (>5000 m^−2^ s^−1^) to determine F_m_. Fluorescence yields were determined as the mean of 5 area readings per fragment. They were measured on the second day of the acclimation period, at the beginning of the heat stress period, and then at 5-day intervals until yields started to decline, upon which measurement frequency was increased to once every 2 to 3 days. During recovery, yields were measured every 6 to 10 days.
3. To assess skeletal growth rates, the buoyant weight of the fragments was measured both before acclimation and after 67 days (during the recovery period), following [45]. Buoyant weights were determined to 0.1 mg with an electronic balance (Shimadzu AW220). To ensure constant seawater density, the same seawater was used for both measurement series and temperature was controlled in a water bath.

### Statistical Analyses

Three sets of analyses were undertaken.

1. Survival was expressed as the proportion of colonies that had survived at the end of the recovery period in each tank, and differences in survival between heat-stressed colonies in response to the four nutrient treatments (Controls, +NO_3_, +OE, +OE +NO_3_) were estimated using a generalized linear model with quasibinomial errors and a logistic link function [46]. Non-significant interactions and main effects were dropped from the models, with only temperature and OE as main effects remaining for *A. millepora* and *M. tuberculosa*, respectively.
2. Trends over time in the fluorescence yields of heat-stressed colonies and differences in these trends in response to the four nutrient treatments were estimated using generalized additive mixed models [47]. The predictors of the models included fixed effects of smooth trends in time and the four nutrient treatments, random effects of tanks and colonies nested in tanks, and first-order autoregressive correlation in time. Based on this model, yields were predicted for the beginning and end of the heat stress period, and the end of the recovery period. Differences in the mean predicted yields due to the four nutrient treatments were estimated for these times. Temporal trends were estimated for each of the treatment groups.
3. Differences in the buoyant weight of colonies between the start of the experiment and the end of the heat stress were estimated using a generalized linear model, with the four nutrient treatments, two temperature levels, and random effects of tanks and colonies nested within tanks as explanatory variables. All statistical analyses used the software package R [48].

## Results

### Survival

**Figure 1:**
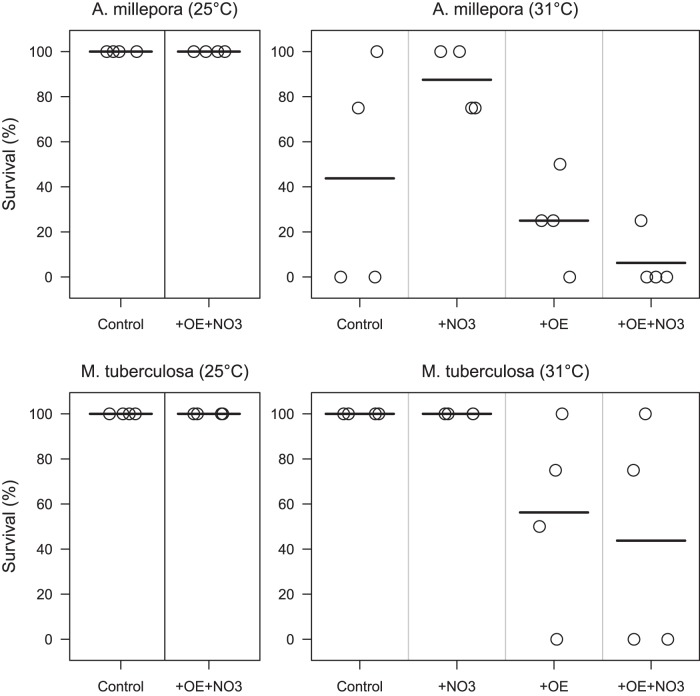
Effects of organic enrichment and nitrate on the survival of heat-stressed and control corals. Survival of *Acropora millepora* (a, b) and *Montipora tuberculosa* (c, d) at the end of the recovery period for the treatments of temperature (25°C (a, c) vs. 31°C (b, d), organic enrichment (+OE), and/or nitrate addition (+NO3; +OE +NO3). The horizontal bars indicate mean percent survival for each treatment; circles mark the percent of surviving colonies for each tank (four colonies per species and tank, four tanks per treatment; points are jittered for clarity).

In the ambient temperature treatments, no mortality was recorded in either species throughout the 90-day long experiment (Fig. 1). Of the heat-stressed *Acropora millepora,* five nubbins (7.8%) died on the last day of the heat stress period (Day 33, 23 days after onset of heat stress), and mortality increased to a mean of 59.4% of colonies per tank by the end of the recovery period. Survival varied greatly between tanks, but means were slightly lower in the two heated +OE treatments compared to those without OE addition (mean survival in the four tanks per treatment: 6.3% and 25% for +OE, vs. 44% and 88%; Fig. 1). The effect of NO_3_ and the interaction between NO_3_ and OE were insignificant, but the difference in mean survival between heated tanks with and without OE was marginally significant (t_15_ = –2.63, P = 0.020).

Of the heat-stressed *Montipora tuberculosa*, none had died after 58 days (48 days of heat stress). In the two treatments without OE, survival remained at 100% throughout the recovery period, in contrast to the two +OE treatments where survival varied widely between tanks but declined to a mean of 44% and 56% at the end of the recovery period (Fig. 1). The difference in mean survival between heated tanks with and without OE tanks was significant (t_15_ = –6.90, P = 0.0009), while the addition of NO_3_ did not affect the survival of heat-stressed *M. tuberculosa*, and there was no interaction between OE and NO_3_.

### Photophysiological Stress and Recovery

**Figure 2:**
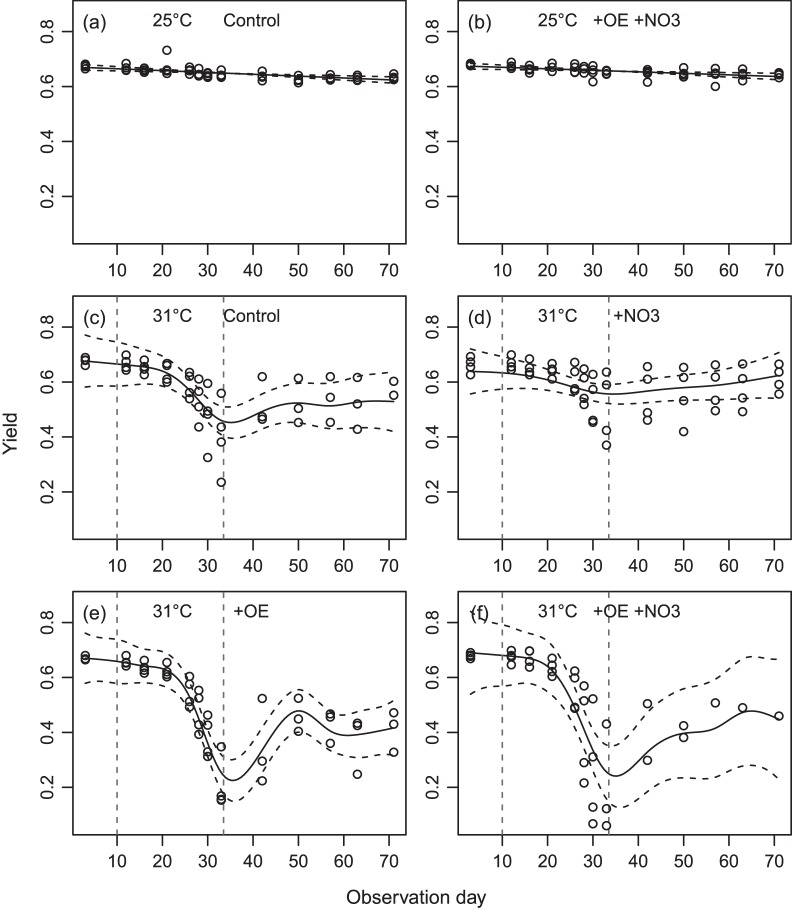
Effects of organic enrichment and nitrate on the fluorescent yields of heat-stressed and control *A. millepora*. Time series of the fluorescence yields in *Acropora millepora.* Two treatments (Controls and +OE +NO3) remained at ambient temperature (mean: 25°C) throughout the experimental period (a, b). Tanks in the other treatments were exposed to heat stress (31.2°C) between Days 10 and 33, followed by a recovery period at ambient temperature (c-f). The nutrient treatments applied to these tanks were (c) Controls, (d) +NO3, (e) +OE and (f) +OE +NO3. Points represent means across colonies for each tank; solid lines are estimated temporal trends and dashed lines are 95% confidence intervals.

**Figure 3:**
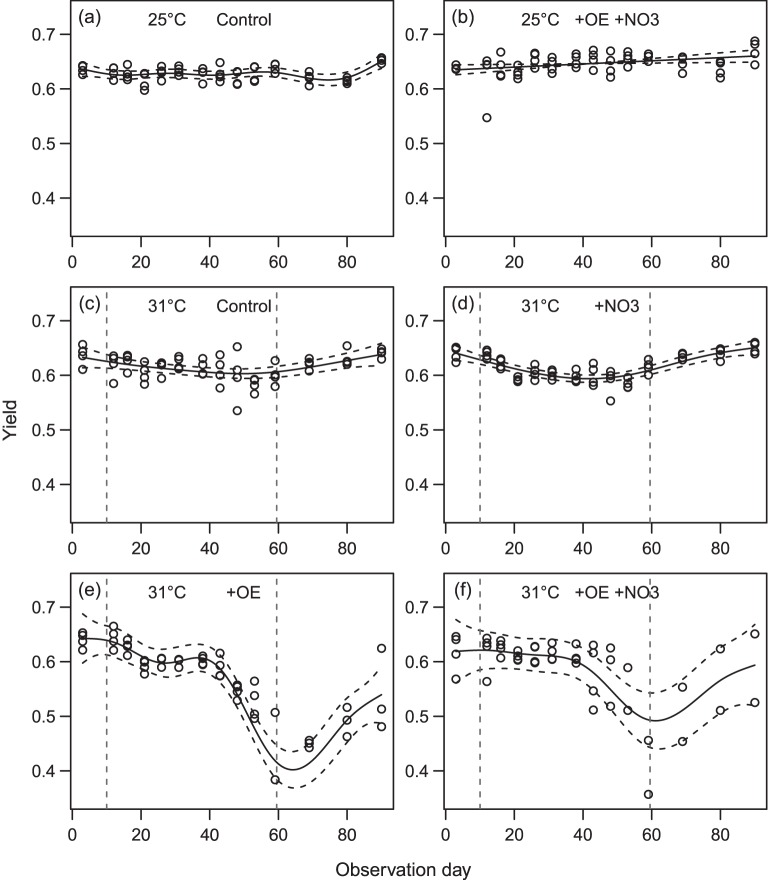
Effects of organic enrichment and nitrate on the fluorescent yields of heat-stressed and control *M. tuberculosa*. Time series of the fluorescence yields in *Montipora tuberculosa.* For details see the legend for Fig. 2. In this species, heat stress (31.2°C) was applied between Days 10 and 59.

Chlorophyll fluorescence yields for each of the species were similar across all treatments at the beginning of the 10-day acclimation period (P>0.05, Figs. 2 and 3). At 25°C, yields of *A. millepora* showed a minor decline (–0.05 units) over time in both +OE +NO_3_ and Controls (Fig. 2a, b), while those of *M. tuberculosa* showed no temporal trend (Fig. 3a, b). After the 10 day acclimatizaton period and for the remaining 60–80 days, tank-averaged yields at 25°C were slightly higher in +OE +NO_3_ compared with controls in both species (*A. millepora*: 0.654±0.018 SD vs. 0.646±0.020, F_(1,88)_ = 6.4, P = 0.01, with also significant differences between tanks; *M. tuberculosa:* 0.647±0.021 vs. 0.628±0.014, F_(1,96)_ = 28.0, P<0.001, no differences between tanks), suggesting a minor photophysiological response to the higher nutrients and slightly reduced light.

*A. millepora* was more susceptible to heat stress than *M. tuberculosa.* Reductions in yields were observed after 13 days of heat stress in *A. millepora,* and after 33 days in *M. tuberculosa* (Figs. 2c–f, and 3c–f). At the end of the heat stress period, F_v_/F_m_ was ≤0.30 in 32.3% and 18.8% of colonies respectively, and these colonies had visibly reduced pigmentation relative to those kept at 25°C. Yields in both *A. millepora* and *M. tuberculosa* were significantly reduced in both +OE treatments, whereas the effects were less severe for tanks without OE (Figs. 2c–f and 3c–f, Tables 2 and 3), despite the 2–3% greater light exposure in the latter.

**Table 2:** Mean fluorescence yields in *Acropora millepora* at the beginning of the experiment (Day 3), at the end of the heat stress (Day 33), and after recovery (Day 71*;* Fig. 2).

	Control		+NO3		+OE		+OE +NO3	
	Yield	SE	Yield	SE	Yield	SE	Yield	SE
Day 3	0.655	0.039	0.620	0.032	0.654	0.038	0.658	0.041
Day 33	0.500	0.021	0.605	0.016	0.290	0.025	0.261	0.024
Day 71	0.531	0.051	0.605	0.036	0.416	0.046	0.399	0.057
Days 33 - 3	**−0.155**	0.044	−0.015	0.035	**−0.364**	0.050	**−0.397**	0.047
Days 71 - 33	0.031	0.055	0.0	0.039	**0.123**	0.052	**0.138**	0.062
Days 71 - 3	−0.124	0.064	−0.015	0.047	**−0.238**	0.063	**−0.259**	0.070

Values are mean yields and SE across four tanks (four colonies per tank) for each of the four heat stressed treatments (31°C). The last three rows show the differences in mean fluorescence yields between days, with significant differences (>2 SE) marked in bold.

**Table 3:** Mean fluorescence yields in *Montipora tuberculosa* at the beginning of the experiment (Day 3), at the end of the heat stress (Day 59), and after recovery (Day 90*;* Fig. 3).

	Control		+NO3		+OE		+OE +NO3	
	Yield	SE	Yield	SE	Yield	SE	Yield	SE
Day 3	0.627	0.013	0.633	0.014	0.640	0.016	0.618	0.016
Day 59	0.611	0.008	0.610	0.009	0.405	0.014	0.484	0.017
Day 90	0.632	0.014	0.647	0.014	0.536	0.019	0.590	0.023
Days 59 - 3	−0.016	0.015	−0.023	0.017	**−0.235**	0.021	**−0.134**	0.024
Days 90 - 59	−0.021	0.016	**−0.038**	0.017	**−0.130**	0.023	**−0.106**	0.029
Days 90 - 3	0.005	0.019	0.015	0.020	**−0.104**	0.025	−0.028	0.028

Values are mean yields and SE across four tanks (four colonies per tank) for each of the four heat stressed treatments (31°C). The last three rows show the differences in mean fluorescence yields between days, with significant differences (>2 SE) marked in bold.

At the end of the recovery period at 25°C, 69.1% and 77.1% of the surviving heat-stressed colonies had yields that had recovered to >0.60 in *A. millepora* and *M. tuberculosa* respectively (Tables 2 and 3). In *A. millepora*, mean yields were still reduced in the +OE treatments at the end of the recovery period (0.24–0.26 units below initial values, Table 2), whereas they were more similar to initial (pre-stress) values in those without OE (–0.12 and –0.02). In *M. tuberculosa*, the recovery was still incomplete in +OE without NO_3_ addition at the end of the experiment (0.1 units below initial values), whereas colonies in the other three treatments had recovered to values similar to their initial values (Table 3).

### Skeletal Growth

**Figure 4:**
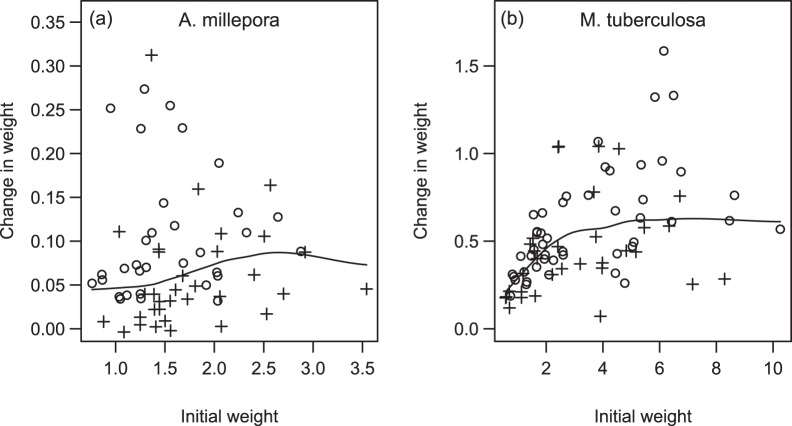
Effects of temperature and organic enrichment on skeletal growth in *A. millepora* and *M. tuberculosa*. Change in weight (g) of surviving colonies over 67 days for (a) *Acropora millepora* and (b) *Montipora tuberculosa*. Fitted grey lines show the relationship between initial weight and weight gain. In *A. millepora,* weight gains were 45% reduced in colonies exposed to 31.2°C (crosses) than those exposed to 25°C (circles), and not related to initial weight. In *M. tuberculosa,* weight gains were 24% reduced in colonies exposed to +OE (crosses) than those not exposed to OE (circles), and declined with initial size for small colony fragments. The other factors under consideration did not significantly affect weight gains in the two species.

Changes in colony weights were calculated, excluding corals that had died. For *A. millepora*, initial and final mean buoyant weights were 1.662 g and 1.743 g respectively, giving a mean gain of 0.081 g. Weight gains were relatively constant across initial weights, but were more variable at low initial weights (Fig. 4a). There were no significant interactions between the effects of temperature, OE and NO_3_, and the main effects of OE and NO_3_ on weight gain were also insignificant. The main effect of temperature was strong, with colonies exposed to heat stress having 45% lower weight gains than those at 25°C (0.058 g *vs* 0.105 g, t_17_ = 2.33, P = 0.032).

For *M. tuberculosa*, initial and final mean weights were 3.377 g and 3.916 g respectively, giving a mean gain of 0.539 g. Weight gains dropped sharply for initial weights <3 g, but plateaued and were more variable at higher weights (Fig. 4b). There were no significant interactions between the effects of temperature, OE and NO_3_, and the main effects of temperature and NO_3_ on weight gain were insignificant. The negative effect of +OE was substantial, with colonies in +OE having 24% reduced weight gains than those without OE (0.453 g vs 0.596 g, t_17_ = 2.12, P = 0.046).

## Discussion

This study demonstrates that exposure to organically and nutrient enriched coastal waters at environmentally relevant concentrations reduces the resistance and resilience of corals to temperature stress, leading to greater reductions in fluorescence yields, lower survival and slower recovery in two common coastal coral species.

There is mounting evidence that heat stress lowers skeletal growth rates, as demonstrated for massive corals such as *Porites* [2], [49], *Montastrea faveolata* [35] and *Diploastrea heliopora* [50], and for the branching coral *Pocillopora damicornis* [51]. For Acroporidae, information on heat stress effects on skeletal growth is still comparatively sparse, except for a field study reporting slightly greater reductions in growth in severely bleached compared to moderately bleached colonies for nine months after a bleaching event [52]. Our observed 45% decline in skeletal growth in heat stressed *A. millepora* therefore adds important information on the potential effects of warming seawater temperatures on *Acropora* growth. This estimate is conservative since the most sensitive individuals had died and were not included in the analysis. In contrast, skeletal growth of the more temperature tolerant *M. tuberculosa* was unaffected by temperature stress. Instead, the growth of this foliose species was reduced by 24% at +OE, potentially reflecting its exposure to settling particles.

Terrestrial runoff, and the associated increase in nutrients and sediments, represents a complex and multi-factorial agent of change, affecting corals through many different but related pathways: (1) increased availability of particulate food, (2) increased exposure to dissolved inorganic nutrients, (3) reduced light availability from reduced water clarity, and (4) increased stress from exposure to organically and nutrient enriched sediments [53]. Particulate and dissolved nutrients, turbidity and sedimentation are often highly correlated, making it difficult to separate their relative influence on organisms and ecosystems [38], [54]. This is due to the rapid uptake of dissolved inorganic nutrients that stimulate succession in phyto- and zooplankton communities, and hence conversion from dissolved inorganic to particulate organic forms [55], [56]. A proportion of these organically enriched particles can serve as food for some species of coral [57], [58]. However, they also reduce water clarity and hence light availability for photosynthesis [59], and stimulate microbial communities, enhancing biological oxygen demand and potentially serve as vector for diseases. Organical enrichment of particulate materials therefore greatly increases the detrimental effects of sedimentation on the photophysiology and survival of juvenile and adult corals [60]–[62]. Organical enrichment can therefore increase both the food availability and stress in corals [57], and shift coral reefs from predominantly phototrophic to more heterotrophic communities [63]. The complexity of terrestrial runoff effects are further increased as they affect corals at several time scales: first, episodic floods expose coastal ecosystems to combined nutrient, pollutant and salinity stress, and second, depending on the geomorphology, bathymetry and currents, through the more chronic enrichment of sediments with organic matter and reduction in water clarity [63]. For example, coastal development and agriculture have led to five- to nine-fold increases in nutrient and sediment discharges into the Great Barrier Reef [41], and these discharges result in not only in high concentrations of nutrients in flood waters, but also prolonged periods of high coastal turbidity during non-flooding times [37], chronically high concentrations of particulate nutrients, but only minor changes in dissolved inorganic nutrients [38].

The strong negative effect of organically and nutrient enriched waters on the corals’ thermal tolerance demonstrated in this study suggests that coastal eutrophication produces an additional stress factor that outweighed nutritional benefits in these two coral species. Interestingly, Anthony et al. [18] showed that exposure to fine particulate matter resuspended from the seafloor around offshore reefs (i. e. similar to our approach, but without nutrient enrichment) resulted in high lipid storage and reduced mortality from temperature stress in *Acropora intermedia* at high irradiance. In contrast to that study, we incubated muddy sediments enriched with nutrients. The difference strongly suggests that it is the organical enrichment that constitutes a stress factor, which simulated the plankton successions in organical and nutrient enriched inshore waters in the field, which contain bacterio-, phyto- and zooplankton, detrital matter, fecal pellets and other organic and inorganic particles colonized by bacteria and microalgae, as well as dissolved organic and inorganic forms of nutrients. Our experimental design simulated natural and environmentally relevant processes, but the trade-off was that its natural complexity does not allow attributing the additional stress to any specific agent. Stress may have been caused by one or several different mechanisms, including: greater oxygen radical production of the dense endosymbionts [25], energetic costs of removing settling particles, oxygen consumption and the release of CO_2_ and metabolic products of the organic-rich materials, altering the seawater chemistry and causing harm when ingested or when settling on colony surfaces [61], [62]. In contrast to the strong effects of organically enriched sediments, the effects of sole provision of dissolved inorganic nitrogen were weak and inconclusive, probably because of rapid uptake, as also observed on eutrophic inshore reefs [53], and despite the apparent nitrogen limited nature of the coastal seawater used.

**Figure 5:**
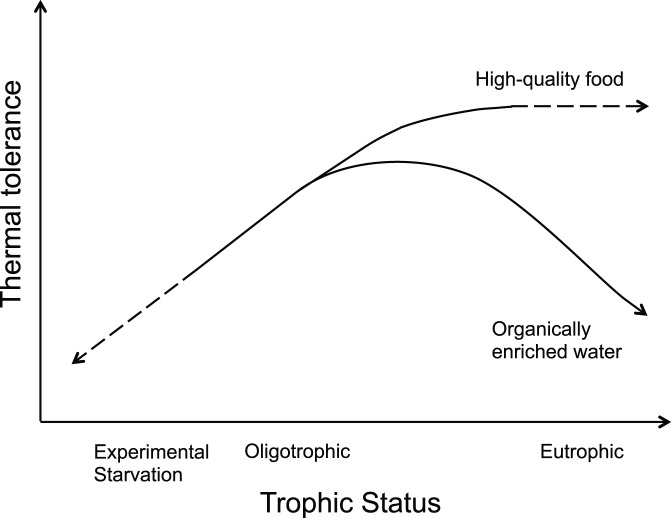
Relationship between nutrient availability and thermal tolerance in corals. The conceptual diagram illustrates the commonly non-linear and divergent relationships between thermal tolerance of heat-stressed coral species and the trophic status of their environment and/or food provision in experiments. See text for definitions and explanations.

Our literature review has shown that both from existing empirical data and theoretical perspective, it has remained surprisingly unclear whether corals exhibit synergistic/additive or antagonistic responses when simultaneously exposed to heat stress and eutrophication. Although the complexity of the water quality problem and the diversity of study methods preclude a quantitative meta-analysis, it allows identifying directions of change along environmental gradients that are consistently observed despite the different study species and methods used. The observed commonalities are summarised in a conceptual trophic framework (Fig. 5), which illustrates two potential reasons for the apparently inconsistent outcomes: First, the strongly non-linear relationship between nutrient (and light) provision and energetic status. It is often forgotten that nutrients and light represent either a stress or a beneficial factor, depending on their levels and on the coral species under investigation. Future studies should therefore focus on testing dose-response relationships using multiple levels of exposure, i.e. regression-based experimental designs rather than contrast-based designs, where it is unknown whether levels are on the left or right side of the response optima. Second, shifts in the trophic status of the environment (from oligotrophic to eutrophic) do not easily translate into shifts in the trophic status of individual reef corals (from starved to well-fed), because the types of food utilized and trophic plasticity vary greatly between species [16], [57], [58]. The diagram illustrates that depending on the quality and concentration of food provided, the exposure to high nutrient conditions may be either beneficial, or may be a stress factor that can be as detrimental as artificial starvation. High quality food includes zooplankton (or *Artemia* in experiments) and other microplankton, and to a more limited extent pico- and nanoplankton and particulate organic matter [58]. Organically enriched and muddy terrestrial runoff enhances all of these types of food, but it also increases the corals’ exposure to indigestible and potentially detrimental materials such as inorganic sediments, refractory detrital material, transparent exopolymer particles, microbial flocs and biofilms, disease-causing microbes and dissolved organic carbon, dissolved inorganic nutrients, and nutrient imbalances. Clearly, more work to understand the mechanisms for the detrimental outcome of exposure to organically rich materials is needed. The comparisons between experimentally starved corals (i.e., deprived of any form of plankton, or stripped of other essential elements such as iron or phosphorus) and those provided with high quality food have been essential to demonstrate the benefits of heterotrophy and the detriment of malnutrition and imbalanced nutrient ratios on thermal tolerance, and their implications for species-specific differences in thermal tolerance. However, our study has shown that some of the findings may not be easily extrapolated to predict bleaching outcomes on reefs that are exposed to high levels of terrestrial runoff of nutrients and sediments, leading to organical enrichment.

In conclusion, this study adds to mounting evidence that eutrophication can worsen thermal stress on inshore reef communities. Even without heat stress, the exposure to organical enrichment has strong negative effects on the photophysiology and survival of inshore corals [60]–[62]. Protecting corals from turbidity, nutrients and sedimentation is not only beneficial for the physiology and survival prospects of existing heat-stressed corals. It also prevents other forms of ecological damage, including declining coral species diversity, increasing macroalgal cover, and increasing frequencies of population outbreaks of the coral eating crown-of-thorns starfish *Acanthaster planci* when exposed to terrestrial runoff [64], [65]. Improving water quality, by reducing the loss of fertilizers and soils from farmed and cleared lands, is therefore rightly considered an essential management strategy to enhance the resilience of reefs to warming temperatures and ocean acidification [8], [9], [43]. Our study re-confirms that the management goal of improving water quality to enhance the resilience of reefs is warranted, as it can improve the thermal tolerance of some corals on coastal coral reefs.
